# Regulation of Nitric Oxide Production in the Developmental Programming of Hypertension and Kidney Disease

**DOI:** 10.3390/ijms20030681

**Published:** 2019-02-05

**Authors:** Chien-Ning Hsu, You-Lin Tain

**Affiliations:** 1 Department of Pharmacy, Kaohsiung Chang Gung Memorial Hospital and College of Medicine, Chang Gung University, Kaohsiung 833, Taiwan; chien_ning_hsu@hotmail.com; 2 Department of Pediatrics, Kaohsiung Chang Gung Memorial Hospital and College of Medicine, Chang Gung University, Kaohsiung 833, Taiwan

**Keywords:** asymmetric dimethylarginine, developmental origins of health and disease (DOHaD), hypertension, kidney disease, nitric oxide, nutrient-sensing signal, oxidative stress, renal programming, renin-angiotensin system, sex differences

## Abstract

Development of the kidney can be altered in response to adverse environments leading to renal programming and increased vulnerability to the development of hypertension and kidney disease in adulthood. By contrast, reprogramming is a strategy shifting therapeutic intervention from adulthood to early life to reverse the programming processes. Nitric oxide (NO) is a key mediator of renal physiology and blood pressure regulation. NO deficiency is a common mechanism underlying renal programming, while early-life NO-targeting interventions may serve as reprogramming strategies to prevent the development of hypertension and kidney disease. This review will first summarize the regulation of NO in the kidney. We also address human and animal data supporting the link between NO system and developmental programming of hypertension and kidney disease. This will be followed by the links between NO deficiency and the common mechanisms of renal programming, including the oxidative stress, renin–angiotensin system, nutrient-sensing signals, and sex differences. Recent data from animal studies have suggested that interventions targeting the NO pathway could be reprogramming strategies to prevent the development of hypertension and kidney disease. Further clinical studies are required to bridge the gap between animal models and clinical trials in order to develop ideal NO-targeting reprogramming strategies and to be able to have a lifelong impact, with profound savings in the global burden of hypertension and kidney disease.

## 1. Introduction

Nitric oxide (NO), a potent vasodilator, plays a crucial role in the regulation of placenta vascular development, feto-placental vascular reactivity, embryogenesis, and fetal development during pregnancy [1,2]. Conversely, maternal NO deficiency relates to compromised pregnancies and adverse fetal outcomes [3,4]. Maternal adverse conditions can affect the structure and function of the fetus that increases the risk of developing chronic diseases in later life. This concept is known as the developmental origins of health and disease (DOHaD) [5].

Non-communicable diseases (NCDs) are the leading cause of global death [6]. Most NCDs are considered preventable as they are caused by modifiable risk factors driven in early life [5]. Hypertension and kidney disease are recognized as major NCDs [6]. Hypertension has a bidirectional relationship with kidney disease: on the one hand it is a major risk factor for initiation and progression of kidney disease and, on the other hand, it is the result of kidney disease itself. According to the DOHaD concept, both disorders may result from early-life insults in nature [7,8]. Despite recent advances in pharmacotherapy and lifestyle modification, there is still a global rising prevalence of both disorders [6]. 

NO production is reduced in hypertension as well as in kidney disease [9,10]. Regulation of blood pressure (BP) is a complicated process that comprises major contributions from the kidney. The developing kidney is vulnerable to a suboptimal in utero environment. Accordingly, suboptimal environments during critical periods of kidney development may produce long-term effects on the kidney by so-called renal programming [11,12,13]. Cumulative evidence implicates the role of the dysregulated NO system in renal programming and in the programming of hypertension [12,13,14,15].

In this review, we discuss the key themes on the impact of NO pathway in the developmental origins of hypertension and kidney disease. We have particularly focused on the following areas: regulation of NO in the kidney; evidence from human studies support fetal programming of hypertension and kidney disease; insight from animal models of renal programming related to the NO pathway; and the application of reprogramming interventions targeting the NO pathway to prevent the programming of hypertension and kidney disease.

## 2. Regulation of Nitric Oxide in the Kidney

NO synthesis occurs via two distinct pathways: nitric oxide synthase (NOS)-independent and NOS-dependent. The NOS-independent pathway involves the reduction of nitrite to NO [16]. This nitrate–nitrite–NO pathway is considered as a complementary pathway to the classical l‑arginine–NOS pathway. There are three NOSs, namely endothelial NOS (eNOS), neuronal NOS (nNOS) and inducible NOS (iNOS), which were thought to be the major intracellular sources of cellular NO. NO is generated from the conversion of l-arginine to l-citrulline by NOS that requires the cofactors tetrahydrobiopterin (BH_4_), flavin adenine dinucleotide, and flavin mononucleotide. Under a physiological state, mainly nNOS and eNOS are constitutively expressed in the kidney, but under a pathological state iNOS is more likely to express [17]. l-arginine supply can be restricted via the arginase enzyme, resulting in NO deficiency. In the depletion of l-arginine or cofactor BH_4_, eNOS uncoupling leads to superoxide production [17]. On the other hand, the kidney can use l-citrulline to make l-arginine via the argininosuccinate (AS) pathway involving AS synthetase and lyase [18].

**Figure 1:**
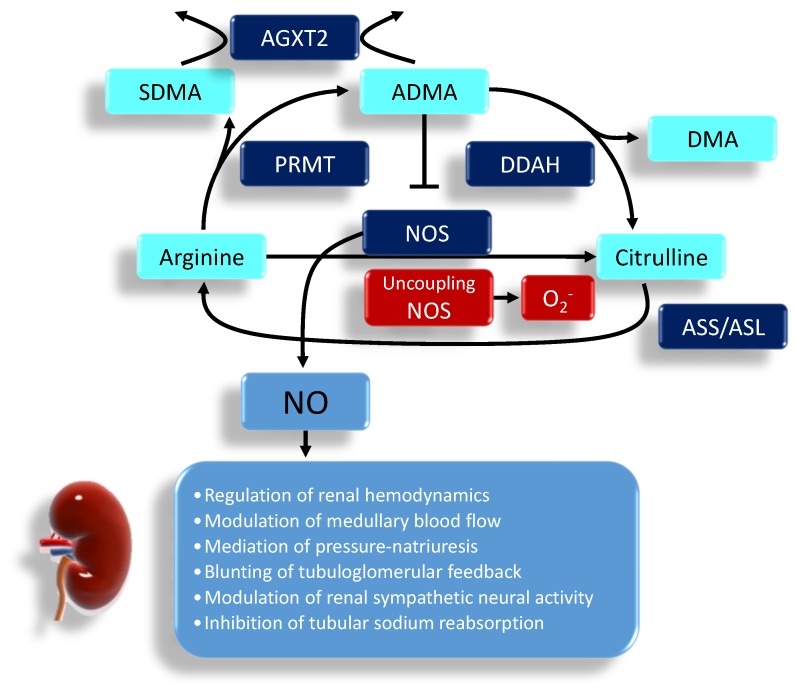
The regulation of the NO system in the kidney. l-arginine has multiple metabolic fates, including metabolism by NOS, arginase, and other enzymes. ADMA is capable of competing with l-arginine to inhibit NO production. Both ADMA and symmetric dimethylarginine (SDMA) come from the methylated l-arginine by protein arginine methyltransferase isoenzyme family (PRMT). Unlike SDMA, only ADMA can be metabolized by dimethylarginine dimethylaminohydrolase (DDAH)-1 and -2 to generate l-citrulline and dimethylamine (DMA). Alanine-glyoxylate aminotransferase 2 (AGXT2) can metabolize ADMA as well as SDMA. On the other hand, l-citrulline can be converted to l-arginine via the argininosuccinate synthetase (ASS) and argininosuccinate lyase (ASL). ADMA can uncouple NOS to produce superoxide. In the kidney, NO is responsible for many physiological functions. The arrow means produces, indicating result of reaction. The T-bar means inhibits.

NO deficiency is also attributed to increased endogenous NOS inhibitors, asymmetric dimethylarginine (ADMA) and symmetric dimethylarginine (SDMA) [19]. ADMA can compete with l-arginine to reduce NOS activity, leading to a decrease of NO. ADMA can also uncouple NOS to produce superoxide, contributing to the burden of oxidative stress [20]. Unlike ADMA, SDMA does not directly inhibit NOS but is a competitive inhibitor of l-arginine transport. Protein-incorporated ADMA is formed by posttranslational methylation: two methyl groups are placed on one of the terminal nitrogen atoms of the quanidino group of arginine in proteins by a family of protein arginine methyltransferases (PRMTs) [19]. The other derivatives, the SDMA, are where one methyl group is placed on each of the terminal guanidino nitrogens. Proteolytic release of free ADMA and SDMA can be moved into or out of cells via the cationic amino acid transporter family. To date, three enzymes have been reported to metabolize ADMA: dimethylarginine dimethylaminohydrolase-1 (DDAH-1) and -2 (DDAH-2) as well as alanine-glyoxylate aminotransferase 2 (AGXT2). Unlike DDAHs, AGXT2, a mitochondrial aminotransferase expressed primarily in the kidney, can metabolize not only ADMA but also SDMA [21]. The biochemical pathways related to the regulation of NO pathway are illustrated in Figure 1. In the kidney, NO has many important functions including the regulation of renal hemodynamics, modulation of medullary blood flow, mediation of pressure-natriuresis, blunting of tubuloglomerular feedback, modulation of renal sympathetic neural activity and inhibition of tubular sodium reabsorption [17]. 

NO deficiency in the kidney can be caused by: (1) l-arginine deficiency, (2) decreased abundance and activity of NOS, (3) inactivation of NO by increased oxidative stress, and (4) increased endogenous NOS inhibitor ADMA. Several lines of evidence indicate that NO deficiency contributes to hypertension and kidney disease [9,10]. First, l-arginine, the substrate for NOS, deficiency is involved in human hypertension and kidney disease [22], but l-arginine supplementation has beneficial effects on BP control [23]. Second, renal nNOS abundance and activity fall with kidney injury that are correlated to decreased NO production and elevation of BP in various rat models of chronic kidney disease [9]. A deficiency of eNOS-derived NO within the kidney exacerbates the damaging effects of diabetic nephropathy in animal models [9,24]. Third are studies of oxidative stress in hypertension and kidney disease [10]. Oxidative stress is mainly caused by an imbalance between the oxidants and antioxidant defense system. Oxidative stress might reduce NO bioavailability by oxidizing cofactor BH_4_ to uncouple NOS, inhibiting DDAH activity to increase ADMA, and scavenging NO by superoxide to form peroxynitrite [25]. Therefore, inactivation of NO by oxidative stress in the kidney may, in part, contribute to the development of hypertension and kidney disease [10,26]. Fourth are reports that increased plasma ADMA levels are associated with hypertension and kidney disease in both humans and animals [27,28]. As aforementioned causes of NO deficiency exist in human trials and experimental studies, these observations support a deficiency of NO in the kidney contributing to the pathogenesis of hypertension and kidney disease. 

## 3. Developmental Programming of Hypertension and Kidney Disease: Insight Provided by Human Study

Important support for the developmental programming of hypertension and kidney disease came from the Dutch famine birth cohort study. Adults exposed to maternal famine developed many disorders, including hypertension and kidney disease [29]. Second are observations that a low nephron endowment is a common denominator underlying the vulnerability to kidney disease and hypertension [30]. Low birthweight (LBW) and prematurity are risk factors for hypertension and kidney disease and both are associated with low nephron number [30,31]. Preterm infants may exhibit low nephron endowment due to a compromised pregnancy, intra-uterine growth retardation (IUGR), inadequacy of postnatal nutrition, and treatment with nephrotoxic medication after birth [31]. A reduced nephron number leads to a higher glomerular capillary pressure and glomerular hyperfiltration. Over time, this process initiates a vicious cycle of rising BP and further nephron loss. A meta-analysis of >2 million individuals reported that those with LBW had a 70% increased risk for development of chronic kidney disease [32]. A case-control study of >1.6 million infants demonstrated that prematurity and LBW are risk factors for congenital anomaly of kidney and urinary tract (CAKUT) [33]. Another line of evidence comes from studies of mother-child cohorts. As reviewed elsewhere [34], several risks affecting early-life BP of offspring in these cohorts include undernutrition, gestational hypertension, maternal obesity, short-term breastfeeding, maternal smoking, low vitamin D intake, and excessive postnatal weight gain. However, these cohorts cannot yet per se directly provide cause-effect relationships between the specific early-life insults and phenotypes in later life. Therefore, it stands to reason that much of our knowledge seems to come largely from animal models to unveil underling mechanisms of renal programming related to hypertension and kidney disease.

## 4. Mechanisms of Renal Programming Related to Nitric Oxide (NO) Pathway

**Figure 2:**
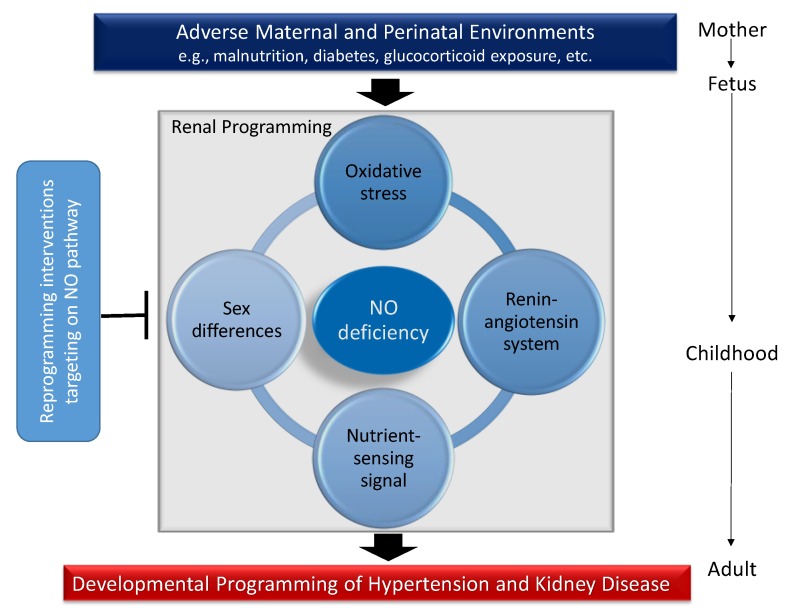
Schema outlining the potential role of NO deficiency on mediating other mechanisms underlying renal programming to cause hypertension and kidney disease in adulthood in response to a variety of early-life insults. Conversely, targeting the NO pathway could be a reprogramming strategy to prevent programmed hypertension and kidney disease by early intervention.

Although several organ systems responsible for BP regulation can be programmed in response to early-life environmental insults, renal programming is considered crucial in the development of hypertension and kidney disease [12,13]. Emerging evidence indicates that there may be common mechanisms underlying renal programming which lead to the pathogenesis of hypertension and kidney disease of developmental origins. Animal models have provided insight on several common mechanisms, including oxidative stress, alterations of renin-angiotensin system (RAS), nutrient-sensing signals, and sex differences [8,12,13,14,15,34]. All of these observations provide a close link between the NO deficiency and other important mechanisms involved in programmed hypertension and kidney disease (Figure 2). Each will be discussed in turn. 

### 4.1. Oxidatice Stress

Oxidative stress is an imbalance between pro-oxidant molecules and antioxidant defenses, mainly related to dysregulation of reactive oxygen species (ROS) and NO. The developing fetus is highly vulnerable to oxidant injury due to its low antioxidant capacity [35]. Thus, early-life NO–ROS imbalance is capable of programming adult hypertension and kidney disease [14,36]. Cumulative evidence indicates that a variety of prenatal insults lead to renal programming and hypertension associated with oxidative stress, including maternal undernutrition [37], maternal diabetes [38], prenatal glucocorticoid administration [39,40,41], preeclampsia [42], and exposure to high-fructose diet [43] and high-fat diet [44] in pregnancy and lactation. Importantly, among these programmed models, the impaired l-arginine–ADMA–NO pathway is closely interrelated to oxidative stress in determining the programming process as we reviewed elsewhere [45].

NO depletion in pregnancy induced by N^G^-nitro-l-arginine-methyl ester (l-NAME, an inhibitor of NOS) caused renal programming, increased oxidative stress, and programmed hypertension in adult offspring [46,47]. Additionally, maternal NO deficiency alters a wide range of signaling pathways as found by the Kyoto Encyclopedia of Genes and Genomes (KEGG) pathway analysis [48]. Among them, the mitogen-activated protein kinases (MAPK) pathway is involved in redox-sensitive signaling, contributing to the development of hypertension [49]. Furthermore, our previous report showed that NO deficiency in embryonic kidneys (metanephroi) induced by ADMA impairs nephrogenesis [50]. Metanephroi grown in 2 or 10 µM ADMA were significantly smaller and contained fewer nephrons in a dose-dependent manner [50]. Metanephroi grown in 10 µM ADMA altered a total of 1221 differential expressed genes by next-generation RNA sequencing (NGS) analysis [51]. Among them, *Avpr1a, Ephx2, Hba2, Hba-a2,* and *Npy1r* have been identified as differentially expressed genes in the kidney in different programmed hypertension models [48,50,51,52]. Thus, results from these studies suggest a link between NO deficiency and oxidative stress in the developmental programming of hypertension and kidney disease. The arrow means produces, indicating result of reaction. The T-bar means inhibits.

### 4.2. Renin-Angiotensin System

The role of RAS in mediating kidney development and regulating BP has received considerable attention [53,54]. Pharmacological blockade of the RAS has been clinically used as the first choice for hypertension and renal protection. This system consists of different angiotensin peptides mediated by distinct receptors. The classic RAS, defined as the angiotensin converting enzyme (ACE)-angiotensin (Ang) II-angiotensin type 1 receptor (AT1R) axis, promotes vasoconstriction and sodium retention. Conversely, the non-classical RAS composed of the ACE2-Ang-(1-7)-Mas receptor axis leads to vasodilatation [54]. The RAS have been reported to be associated with developmental programming of hypertension in a variety of models, including prenatal glucocorticoid administration [39,40,41], high-fat diet [44], low-protein diet [55], high-sucrose diet [56], and high-fructose diet [57]. NO inhibition by L-NAME in pregnancy caused programmed hypertension in adult offspring, which was associated with increased mRNA of renin and ACE in offspring kidney [47]. On the other hand, blockade of the classical RAS between 2–4 weeks of age has been reported to prevent the developmental programming of hypertension [57,58,59,60]. These protective effects are not only directed upon the RAS, but also through regulating the NO system. In spontaneously hypertensive rat (SHR), early therapy with aliskiren, a renin inhibitor, has been reported to reduce ADMA, restore l-arginine-to-ADMA ratio, and increase renal cortical nNOS protein level to prevent the development of hypertension [61]. Similarly, early aliskiren therapy protects adult rat offspring exposed to maternal caloric restriction against programmed hypertension via ADMA reduction [60]. Nevertheless, the detailed mechanisms underlying the interplay between the RAS and NO pathway contributing to the programmed hypertension and kidney disease need to be further investigated.

### 4.3. Nutrient-Sensing Signals

Nutrient-sensing signals play a crucial role in fetal metabolism and development. Imbalanced nutrition and metabolic status during pregnancy can disturb nutrient-sensing signals, resulting in renal programming and developmental hypertension [45,61]. Several well-known nutrient-sensing signaling pathways exist in the kidney, including cyclic adenosine monophosphate (AMP)-activated protein kinase (AMPK), silent information regulator transcript (SIRT), peroxisome proliferator-activated receptors (PPARs), and PPARγ coactivator-1α (PGC-1α) [62]. The interplay between AMPK and SIRTs, driven by maternal nutritional interventions were found to regulate PPARs and their target genes, thereby driving a programmed process of hypertension [45,63]. Among the PPAR target genes [64], *Nos2, Nos3, Sod2*, and *Nrf2* are related to NO pathway and oxidative stress. AMPK, SIRT1, and PGC-1α can also promote autophagy, a lysosome-mediated degradation process for damaged cellular constituents [65]. Since eNOS-derived NO is capable to activate PGC-1α via AMPK to regulate mitochondrial biogenesis [66], the interplay between NO and nutrient-sensing signals tightly controls the mitochondrial lifecycle (mitochondrial biogenesis vs. removal by autophagy) [67]. 

AMPK activators and PPAR modulators have been proposed as reprogramming strategies for programmed hypertension and kidney disease [63,68]. Using a combined maternal plus post-weaning high-fat diet model, we found that resveratrol, an AMPK activator, prevents the two-hit induced hypertension and increases protein levels of SIRT1, AMPK2α, and PGC-1α in the offspring kidney [69]. Also, resveratrol reduces renal ADMA concentration as well as oxidative stress damage. These results provide evidence for the contribution of nutrient-sensing signals in renal programming and thus for the development programming of hypertension.

### 4.4. Sex Differences

Sex differences in the developmental programming of kidney disease and hypertension have been reported [13,70,71], showing that males are more vulnerable than females. Indeed, several common mechanisms of renal programming, such as the oxidative stress [72], RAS [73] and nutrient-sensing signal [74] have been documented a sex-specific response to environmental insults. The renal transcriptome in response to early-life stimuli is also sex-specific [57,75,76]. Our previous report documented that maternal high-fructose diet induced sex-specific alterations of renal transcriptome [57]. At one week of age, maternal high-fructose consumption caused greater changes of renal transcriptome in female offspring than male offspring [57]. Our finding is in agreement with another study showing that more genes in the placenta were affected in females than in males under different maternal diets [77]. Whether the increased female sensitivity to maternal diet might buffer the deleterious programming effects to protect the female fetuses, leading to a better adaptation and less impact of programmed hypertension and kidney disease in adulthood awaits further evaluation. It is noteworthy that NO production is better preserved in females than in males [78]. The mechanisms responsible for these sex differences in programmed hypertension and kidney disease are not well understood. Thus, better understanding of the impact of NO system on sex-dependent mechanisms that underlie renal programming will aid in developing novel sex-specific strategies to prevent programmed kidney disease and hypertension in both sexes.

### 4.5. Others 

There are other potential mechanisms related to renal programming by which NO signaling might act: (1) sodium transporters, (2) epigenetic regulation, and (3) gut microbiota. First, hypertension and kidney disease have been associated with increased expression/activity of sodium transporters and enhanced sodium reabsorption [8,15]. NO has an inhibitory effect on the activity of several sodium transporters [79]. Thus, it is speculated that NO deficiency may fail to counterbalance the impaired sodium transporters induced by early-life insults, thus leading to programmed hypertension. Next, epigenetic regulation such as histone modifications, DNA methylation, and non-coding RNAs are involved in developmental programming [80]. Histone deacetylases have been reported to epigenetically regulate several genes belonging to the RAS [81]. Although NO has been considered as an epigenetic modulator, the epigenetic effects of NO on the aforementioned mechanisms have not been pursued in animal models of development programming to any great extent. Moreover, emerging evidence documents that the development of hypertension is correlated with gut microbiota dysbiosis [82,83]. Of note, inhibition of NO is proposed as a potential mechanism linking dysbiosis and hypertension [82]. Thus, additional studies are required to elucidate whether early-life gut microbiota dysbiosis may elicit adverse effects on renal programming leading to hypertension and kidney disease in adulthood via regulation NO pathway. 

## 5. Reprogramming Interventions Targeting the NO Pathway to Prevent the Programming of Hypertension and Kidney Disease

Reprogramming strategies targeting the NO pathway to reverse the programming processes that have been employed in a variety of animal models of programmed hypertension and kidney disease, some of which are listed in Table 1 [38,41,46,60,61,84,85,86,87,88,89,90,91,92,93,94,95,96,97,98,99,100,101]. This list is by no means complete and is expected to grow rapidly as the field of DOHaD research flourishes. Currently, a variety of therapeutic interventions have been reported for prevention of programmed hypertension and kidney disease, such as supplementation of NO substrate, ADMA-lowering agents, NO donors, and enhancement of the expression and/or activity of NOS.

In the current review, limited information is available about the use of large animals to study the role of NO on developmental programming of hypertension and kidney disease. As shown in Table 1, rats are the most commonly used among the small animal models. Rats grow rapidly in childhood and reach sexual maturity after six weeks. In adulthood, one rat month is comparable to three human years [102]. Accordingly, Table 1 lists the protective effects of interventions on hypertension and kidney disease evaluated in rodents with different ages, which allows calculations to extract the information that can be translated to humans of a specific age group. 

Next, all of the studies listed in Table 1 used the tail cuff method for the measurement of BP. Since conscious BP measurements by tail cuff might be influenced by stress and show discrepancies with telemetry and indwelling catheters, additional studies are needed to validate BP measurement with the other methods. There are numerous early-life insults inducing developmental programming of hypertension and kidney disease, such as maternal diabetes [38,84], maternal caloric restriction [60,88], maternal NO deficiency [46], prenatal dexamethasone exposure [41], prenatal dexamethasone plus TCDD exposure [91], maternal high-fructose diet plus post-weaning high-salt diet [92], and prenatal dexamethasone plus postnatal high-fat diet [93,94]. Reprogramming effects of interventions targeting the NO pathway to prevent hypertension and kidney disease of developmental origins have been reported ranging from 9 to 50 weeks of rat age. Each of the aforementioned interventions targeting the NO pathway will be discussed in the following section.

### 5.1. Substrate for Nitric Oxide Synthase (NOS)

l-arginine supplementation is widely used to generate NO in experimental studies [103]. Post-weaning supplementation with l-arginine, the substrate for NOS, has been reported to prevent the development of hypertension in a maternal streptozotocin-induced diabetes model [84]. In genetic hypertension rat models, early supplementation of l-arginine and antioxidant starting from prehypertensive stage can protect spontaneously hypertensive rats (SHR) and fawn-hooded hypertensive rat (FHH) against hypertension in adulthood [85,86,87]. However, l-arginine is not a good NO precursor due to its multiple metabolic fates. l-citrulline is the precursor of l-arginine. Unlike l-arginine, it bypasses hepatic metabolism, it is not a substrate of arginase, and it does not induce adverse effects of l-arginine [104]. Approximately 60% of de novo l-arginine synthesis occurs in the kidney, where l-citrulline is taken up and metabolized into l-arginine by the action of argininosuccinate synthetase and argininosuccinate lyase [105]. Thus, supplemental l-citrulline has promise as a therapeutic intervention in many adult diseases related to NO deficiency [104]. Maternal supplementing with l-citrulline in pregnancy and lactation protects adult offspring against the developmental programming of hypertension induced by maternal caloric restriction [88], NO inhibition [46], maternal diabetes [38], and prenatal dexamethasone exposure [41]. Additionally, early l-citrulline supplementation in the pre-hypertensive stage blocks the development of hypertension in SHRs [89,90]. Therefore, a better understanding of the protective effects of substrates for NOS underlying programmed hypertension and kidney disease is warranted.

### 5.2. Asymmetric Dimethylarginine (ADMA)-Lowering Agents

As reviewed elsewhere [14,19,106], a lot of currently used drugs have been reported to lower ADMA levels and restore NO-ROS balance in human and experimental studies. Telmisartan, resveratrol, melatonin, atorvastatin, N-acetylcysteine, vitamin E, salvianolic acid A, oxymatrine, metformin, and rosuvastatin can increase the activity and/or expression of DDAHs (ADMA-metabolizing enzymes) and thereby reduce ADMA levels [14]. On the other hand, telmisartan, rosuvastatin, glucagon-like peptide-1 receptor agonist, and epigallocatechin-3-gallate have been reported to reduce ADMA levels via decreased PRMT-1 (ADMA-generating enzyme) expression. However, only few ADMA-lowering agents have been examined in the developmental programming models to prevent hypertension and kidney disease. 

Maternal treatment with resveratrol, melatonin, or N-acetylcysteine has been reported to reduce plasma ADMA level and protect adult offspring against programmed hypertension in different two-hit models [91,92,93]. Additionally, our previous report demonstrated that early blockade of the RAS by aliskiren offsets the effects of maternal caloric restriction-induced programmed hypertension, which is related to the reduction of ADMA levels [60]. Moreover, early treatment with dimethyl fumarate, a nuclear factor erythroid-derived 2-related factor 2 (Nrf2) activator prevents prenatal dexamethasone and postnatal high-fat diet induced programmed hypertension in male offspring [94]. One of the beneficial effects of dimethyl fumarate treatment is via decreasing plasma ADMA level. Similar to programming hypertension models, aliskiren [61], metformin [95], melatonin [96], and N-acetylcysteine [97] have been demonstrated to block the development of hypertension in SHRs by decreasing plasma ADMA levels. 

Nevertheless, so far, a specific ADMA-lowering agent remains unreachable in clinical practice. Since PRMTs are responsible for the generation of ADMA, and that DDAHs and AGXT2 regulate its metabolism, the discovery of specific PRMT inhibitors, DDAHs agonists, and AGXT2 activators might aid in developing a therapeutic approach to lower ADMA and restore NO, and thereby prevent the development of hypertension and kidney disease for clinical translation.

### 5.3. NO Donors

Nitrate and nitrite are the main substrates to produce NO via the NOS-independent pathway [16]. Our previous study showed that dietary supplementation of nitrate, in amounts resembling a rich intake of vegetables in humans, is able to prevent the development of hypertension in young SHRs [89]. Additionally, two NO donors, molsidomine and pentaerythritol tetranitrate have been reported to prevent the development of hypertension in FFH rats and SHRs, respectively [98,99]. However, nearly no studies to date have tested NO donors in programming models to prevent hypertension and kidney diseease of developmental origins. 

### 5.4. Others

Enhancement of NOS expression and/or activity is another way to increase NO production. The N-terminus of nNOS could bind to a protein named protein inhibitor of nNOS (PIN). Binding of PIN destabolizes nNOS dimers and inhibits nNOS activity, thereby reducing NO production [107]. We previously demonstrated that renal PIN expression is increased in pre-hypertensive and hypertensive stages in SHRs. While inhibition of PIN expression by short interfering RNA targeting PIN attenuates the development of hypertension in SHRs at 12 weeks of age [100]. On the other hand, supplementaing melinjo (*Gnetum gnemon*) seed extract during lactation has been shown to protect adult female offspring against maternal high-fructose diet-induced hypertension via enhancing eNOS expression [101].

## 6. Conclusions

Despite recent advances in pharmacotherapies for hypertension and kidney disease, only a few studies have targeted their potential for reprogramming. Adult hypertension and kidney disease can originate in early life. This concept opens a new window for preventing the development of hypertension and kidney disease via a reprogramming strategy. This review has provided an overview on the various reprogramming strategies that are relevant to the NO pathway, including substrates for NOS, NO donors, ADMA-lowering agents, and enhancement of NOS expression and/or activity. Although emerging evidence from animal studies supports NO as a reprogramming strategy for long-term protection against hypertension and developmental kidney disease, these results await further clinical translation. In the current review, the beneficial effects of these NO-targeting interventions are all coming from small animals. There remains a lack of data regarding large animal models that can allow for the translation of basic science into clinical therapies. This is of growing importance because targeting the NO pathway as a reprogramming strategy against renal programming is a flourishing field and will become even more important in light of the rising epidemic of hypertension and kidney disease. 

## Figures and Tables

**Table 1:** Reprogramming strategies targeting the NO pathway to prevent hypertension and kidney disease of developmental programming in animal models.

Interventions	Animal Models	Intervention Period	Species/Gender	Age at Measure (Week)	Protective Effects	Reference
Substrate for NOS						
l-arginine	Maternal streptozotocin-induced diabetes	3 weeks to 24 weeks	Wistar/M	24	Prevented hypertension and glomerular hypertrophy	[84]
l-arginine + antioxidants	Genetic hypertension	2 weeks before until 8 weeks after birth	SHR/Mand F	9	Prevented hypertension	[85]
l-arginine + antioxidants	Genetic hypertension	2 weeks before until 4 weeks after birth	FHH/M and F	36	Prevented hypertension, proteinuria, and glomerulosclerosis	[86]
l-arginine + antioxidants	Genetic hypertension	2 weeks before until 8 weeks after birth	SHR/M and F	50	Prevented hypertension and proteinuria	[87]
l-citrulline	Maternal 50% caloric restriction	3 weeks before until 3 weeks after birth	SD/M	12	Prevented kidney damage, increased nephron number	[88]
l-citrulline	Maternal nitric oxide deficiency	3 weeks before until 3 weeks after birth	SD/M	12	Prevented hypertension	[46]
l-citrulline	Maternal streptozotocin-induced diabetes	3 weeks before until 3 weeks after birth	SD/M	12	Prevented hypertension and kidney damage, increased nephron number	[38]
l-citrulline	Prenatal dexamethasone exposure	3 weeks before until 3 weeks after birth	SD/M	12	Prevented hypertension, increased nephron number	[41]
l-citrulline	Genetic hypertension	4 weeks to 12 weeks	SHR/M	12	Prevented hypertension	[89]
l-citrulline	Genetic hypertension	2 weeks before until 6 weeks after birth	SHR/M and F	50	Prevented hypertension	[90]
Asymmetric dimethylarginine (ADMA)-lowering agents						
Resveratrol	Prenatal dexamethasone plus TCDD exposure	3 weeks before until 3 weeks after birth	SD/M	12	Prevented hypertension	[91]
Melatonin	Maternal high-fructose diet plus post-weaning high-salt diet	3 weeks before until 3 weeks after birth	SD/M	12	Prevented hypertension	[92]
Aliskiren	Maternal caloric restriction	2 weeks to 4 weeks	SD/M	12	Prevented hypertension	[60]
*N*-acetylcysteine	Prenatal dexamethasone plus postnatal high-fat diet	3 weeks before until 3 weeks after birth	SD/M	16	Prevented hypertension	[93]
Dimethyl fumarate	Prenatal dexamethasone plus postnatal high-fat diet	3 weeks to 6 weeks	SD/M	16	Prevented hypertension	[94]
Metformin	Genetic hypertension	4 weeks to 12 weeks	SHR/M	12	Prevented hypertension	[95]
*N*-acetylcysteine	Genetic hypertension	4 weeks to 12 weeks	SHR/M	12	Prevented hypertension	[96]
Melatoin	Genetic hypertension	4 weeks to 12 weeks	SHR/M	12	Prevented hypertension	[97]
Aliskiren	Genetic hypertension	4 weeks to 12 weeks	SHR/M	12	Prevented hypertension	[61]
NO donor						
Nitrate	Genetic hypertension	4 weeks to 12 weeks	SHR/M	12	Prevented hypertension	[89]
Molsidomine	Genetic hypertension	2 weeks before until 4 weeks after birth	FHH/M and F	42	Prevented hypertension	[98]
Pentaerythritol tetranitrate	Genetic hypertension	3 weeks before until 3 weeks after birth	SHR/M and F	24	Prevented hypertension	[99]
Others						
Short interfering RNA targeting PIN	Genetic hypertension	4 weeks to 12 weeks	SHR/M	12	Prevented hypertension	[100]
Melinjo (Gnetum gnemon) Seed Extract	Maternal high-fructose diet	Birth to 3 weeks	Wistar/F	17	Prevented hypertension	[101]

Studies tabulated according to types of intervention, animal models and age at measure. TCDD = 2,3,7,8-tetrachlorodibenzo-p-dioxin. SD = Sprague–Dawley rat. SHR = spontaneously hypertensive rat. FHH = Fawn-hooded hypertensive rat. M = male. F = female. PIN = protein inhibitor of neuronal nitric oxide synthase.

## Abbreviations

ADMA	Asymmetric dimethylarginine
AGXT2	Alanine-glyoxylate aminotransferase 2
AMPK	Adenosine monophosphate-activated protein kinase
CAKUT	Congenital anomaly of kidney and urinary tract
CAT	Cationic amino acid transporter
DDAH	Dimethylarginine dimethylaminohydrolase
DOHaD	Developmental origins of health and disease
FHH	Fawn hooded hypertensive rat
KEGG	Kyoto Encyclopedia of Genes and Genomes
L-NAME	N^G^-nitro-L-arginine-methyl ester
NCD	Non-communicable disease
NOS	Nitric oxide synthase
PGC-1α	Peroxisome proliferator-activated receptor-γ coactivator-1α
PPAR	Peroxisome proliferator-activated receptor
PIN	Protein inhibitor of neuronal nitric oxide synthase
PRMT	Protein arginine methyltransferase
RAS	Renin-angiotensin system
SDMA	Symmetric dimethylarginine
SHR	Spontaneously hypertensive rat
SIRT	Silent information regulator transcript
TCDD	2,3,7,8-tetrachlorodibenzo-p-dioxin
